# Transcriptome signature of the adult mouse choroid plexus

**DOI:** 10.1186/2045-8118-8-10

**Published:** 2011-01-18

**Authors:** Fernanda Marques, João C Sousa, Giovanni Coppola, Fuying Gao, Renato Puga, Helena Brentani, Daniel H Geschwind, Nuno Sousa, Margarida Correia-Neves, Joana A Palha

**Affiliations:** 1 Life and Health Sciences Research Institute (ICVS), School of Health Sciences, University of Minho, Campus Gualtar, 4710-057 Braga, Portugal; 2 Program in Neurogenetics, Department of Neurology, David Geffen School of Medicine-UCLA, Los Angeles, CA, 90095-1761, USA; 3 Hospital A. C. Camargo, São Paulo, SP, 01509-010, Brazil

## Abstract

**Background:**

Although the gene expression profile of several tissues in humans and in rodent animal models has been explored, analysis of the complete choroid plexus (CP) transcriptome is still lacking. A better characterization of the CP transcriptome can provide key insights into its functions as one of the barriers that separate the brain from the periphery and in the production of cerebrospinal fluid.

**Methods:**

This work extends further what is known about the mouse CP transcriptome through a microarray analysis of CP tissue from normal mice under physiological conditions.

**Results:**

We found that the genes most highly expressed are those implicated in energy metabolism (oxidative phosphorylation, glycolysis/gluconeogenesis) and in ribosomal function, which is in agreement with the secretory nature of the CP. On the other hand, genes encoding for immune mediators are among those with lower expression in basal conditions. In addition, we found genes known to be relevant during brain development, and not previously identified to be expressed in the CP, including those encoding for various axonal guidance and angiogenesis molecules and for growth factors. Some of these are known to influence the neural stem cell niche in the subventricular zone, highlighting the involvement of the CP as a likely modulator of neurogenesis. Interestingly, our observations confirm that the CP transcriptome is unique, displaying low homology with that of other tissues. Of note, we describe here that the closest similarity is with the transcriptome of the endothelial cells of the blood-brain barrier.

**Conclusions:**

Based on the data presented here, it will now be possible to further explore the function of particular proteins of the CP secretome in health and in disease.

## Background

The choroid plexus (CP) is located in the ventricles of the vertebrate brain. It is formed by a monolayer of epithelial cells that surrounds a central stroma in which fenestrated blood vessels are embedded in a rich extracellular matrix. In addition, and depending on the physiological or pathological conditions, the stroma also contains cells such as fibroblasts, macrophages, neutrophils, dendritic cells, plus B and T cells. Tight junctions between the apical sides of the epithelial cells separate the blood from the cerebrospinal fluid (CSF); this provides the barrier function that normally hinders the free movement of molecules and cells from the blood into the CSF. It is well recognized that the CP's principal function is the production of CSF [1]. However, it is also known that the CP participates in other key functions, namely the transport of nutrients into the brain and the removal of metabolic products out of the brain [2]. This seems to be of relevance in diseases such as Alzheimer's, where CP proteins in the apical membrane participate in the clearance of the amyloid beta peptide, alone or bound to CSF carrier proteins [3-5]. In addition, the CP is able to respond to challenges by displaying additional functions not present under basal conditions. It has been recently shown that the CP specifically responds differently to peripheral inflammation, depending on whether the inflammation is acute or sustained, by secreting several immuno modulators [6-9]. Moreover, in conditions of inflammation, the CP seems to be important in the regulation of brain iron homeostasis [10,11]. The CP is also one of the first places of entry for the immune cells during neurological disorders, as shown in the experimental autoimmune encephalomyelitis model of multiple sclerosis [12].

Although a vast number of CP mRNA/proteins have been previously described in the literature, discussion of the full basal transcriptome is still lacking. The data herein presented supports the view that the CP is more than a barrier that restricts the passage of molecules and cells in and out of the brain, but rather a tissue that should be better studied as an active participant in brain homeostasis in both physiological and pathological conditions.

## Methods

All experiments and procedures followed the European Community Council Directive 86/09/EEC guidelines for the care and handling of laboratory animals and were approved by the Life and Health Sciences Research Institute's ethical committee and by the Portuguese Veterinary Authorities ("Direcção Geral de Veterinária" process reference number 520/000/000/2006).

### Animals

All experiments were conducted using 8- to 9-week old C57BL/6 male mice (Charles River, Barcelona, Spain). Animals were maintained under 12 h light/dark cycles at 22.5°C and 55% humidity and fed with regular rodent chow and tap water *ad libitum*. In order to reduce the stress-induced changes in the hypothalamus-pituitary axis, animals were handled for 1 week prior to the start of the experiment. Mice were anesthetized with intraperitoneal injection of ketamine hydrochloride (150 mg/Kg) plus medetomidine (0.3 mg/Kg) and transcardially perfused with cold saline. The CPs were rapidly removed, stored in RNA later (Ambion, Austin, TX, USA) and kept at -80°C. CP isolation was made under conventional light microscopy (SZX7, Olympus, Hamburg, Germany). Three pools of CP (each containing tissue removed from the 4 ventricles of 3 mice) were prepared at the time of sacrifice. While most of the cells are CP epithelial cells, the tissue sampled may also contain stroma cells (e.g. endothelial cells and pericytes). These animals correspond to those used as controls (basal transcriptome) in Marques *et al*. [8].

### Microarray experimental design and data analysis

Total RNA was isolated with Trizol (Invitrogen, Carlsbad, CA, USA) following manufacturer's instructions. After quality assessment using the Agilent Bioanalyzer (Agilent Technologies, CA, USA), 100 ng of total RNA from each pool was amplified and labelled with Illumina TotalPrep RNA Amplification Kit (Illumina Inc., San Diego, CA, USA). The labelled cRNA was then hybridized in a total of two Illumina Whole-genome Mouseref-8 expression Beadchips (Illumina Inc.), following the recommended protocol, each containing 8 arrays, comprising a total of 24,049 well-annotated RefSeq transcripts per beadchip.

After scanning, raw data from BeadStudio software (Illumina Inc.) was read into R/Bioconductor and normalized using quantile normalization. A linear model was applied to the normalized data using Limma package in R/Bioconductor [13]. A contrast analysis was applied and differentially expressed genes were selected using a Bayesian approach with a false discovery rate of 5%. All data is Minimum Information About a Microarray Experiment (MIAME)-compliant and the raw data has been deposited in the GEO database (accession: GSE23714). In order to analyze and compare the CP transcriptome with transcriptomes from other tissues, the initial list containing the 24,049 CP genes was filtered: the repeated genes present in the array platform were excluded and the genes with a Coefficient of Variation (CV) ≥ 30 across the 3 replicates were also removed. The final gene list included 18,160 genes. For the analysis, normalized genes were categorized using the Gene Set Analysis Toolkit from WebGestalt (http://bioinfo.vanderbilt.edu/webgestalt/[14]) or the Ingenuity tools (Redwood City, CA, USA). Enrichment analysis was performed using DAVID (http://david.abcc.ncifcrf.gov/[15]) and Ingenuity (http://www.ingenuity.com[16]) software. For comparison with other transcriptomes, two studies of the GEO database were used: one containing 61 mouse different tissues in physiological conditions (GDS592) [17], and another containing the transcriptome of the PECAM-1+ blood-brain barrier (BBB) microvascular endothelial cells (ppMBMECs) (GSE14375) [18]. From each gene list the repeated genes and the genes with CV ≥ 30 were removed. After these corrections, a list containing the genes common in the three studies was created, analyzed with the Limma package to normalize data between arrays and, subsequently, a cluster was constructed using the Multiexperiment Viewer program.

The GEO database has data on two other CP studies. One focuses on the differential CP expression between B10.pl WT and B10.PL RAG-/- mice (GSE11443); the other evaluates the gene expression profile of 24 neural tissues (including the CP from the 4^th ^ventricle) and 10 other body tissues/organs (GSE3594). We compared our transcriptome with the control transcriptome of each of these two other studies. For these comparisons, we selected the common genes excluding those presenting a CV ≥ 30. After these corrections, data between arrays was normalized with the Limma package, followed by a comparison of the expression level for each gene.

### Confirmation of the array data by RT-qPCR

In order to validate the array data, 20 genes within the lower range of expression (6.1-7.0) were analyzed in an independent set of CP samples. The selected genes were: inducible T-cell co-stimulator, myotubularin-related protein 7, retinal pigment epithelium 65, dual oxidase 2, arginine vasopressin receptor 1B, glutathione peroxidase 5, nestin, epithelial mitogen, interleukin 6, neuropeptide Y, matrix metalloproteinase 9, glucagon receptor, claudin 15, ceruloplasmin, lipocalin 2, CD14 antigen, vav 1 oncogene, retinoic acid receptor gamma, interferon regulatory factor 1 and toll-like receptor 4. Gene abbreviations and gene names are specified in accordance with the HUGO Gene Nomenclature Committee at http://www.genenames.org/[19]. Total RNA was isolated from five pools of CP using Trizol reagent (Invitrogen), and 500 ng amplified using the Superscript RNA amplification system (Invitrogen) according to the manufacturer's instructions. CP RNA was reverse transcribed using random primers of the Superscript First-strand Synthesis system for RT-PCR (Invitrogen).

The oligonucleotide primers (sequences available upon request) were designed using the Primer3 software on the basis of the GenBank sequences. Real-time PCR reactions, using equal amounts of total RNA from each sample, were performed on a CFX 96™ real-time system instrument (Bio-Rad Laboratories, Hercules, CA, USA) using QuantiTect SYBR Green RT-PCR reagent kit (Qiagen, Hamburg, Germany). Product fluorescence was detected at the end of the elongation cycle. All melting curves exhibited a single sharp peak at a temperature characteristic of the primers used.

### Construction of biological function networks

In order to identify major functions of the CP, networks based on the genes displaying higher expression (threshold 13 in the array) were constructed. Analysis yielded 59 genes that were subsequently analysed in the FunNet (http://www.funnet.info[20]) for GO Molecular Function and Kyoto Encyclopedia of Genes and Genomes (KEGG) functional networks. This program clusters the genes depending on their biological function, creating nodes corresponding to those that are most enriched. The relation between every pair of genes is calculated using the signal expression of the genes on the corresponding biological functions.

## Results

### CP transcriptome characterization

**Figure 1:**
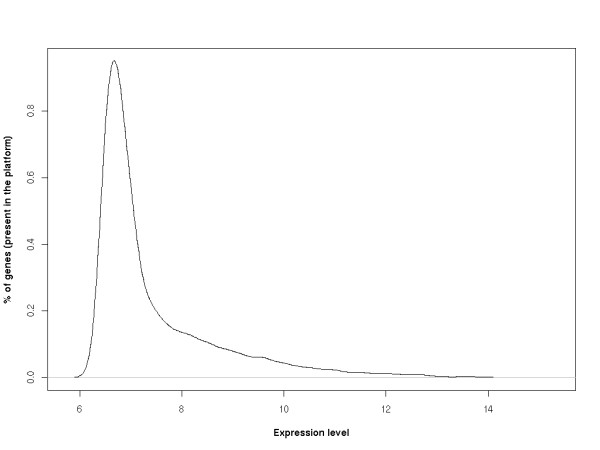
**Frequency graph showing choroid plexus gene expression profile**. From the 18,160 analyzed genes, approximately 81% of the genes presented with expression levels between 6.1 and 8.3; 15% were expressed at levels between 8.3-10.5; 4% were expressed with the level between 10.5-12.8 and only 0.4% of the genes were highly expressed by the CP and presented with an expression level between 12.8-15.0.

From the complete array analysis, we found that the majority of genes in the CP transcriptome are expressed at very low levels. From a range of expression levels that varied from 6.1-15.0, (log2-transformed absolute expression levels), 81% of the genes were expressed between the 6.1-8.3 level; 15% were expressed at levels between 8.3-10.5; 4% were expressed with the level between 10.5-12.8 and only 0.4% of the genes were highly expressed and presented an expression level between 12.8-15.0 (Figure 1). All the genes detected and their relative expression levels are presented as Additional File 1 (Expression levels of the genes transcribed in the basal choroid plexus) and deposited in the GEO database GSE23714.

Twenty genes, with expression levels ranging from 6.1 to 7.0, were further analyzed by RT-qPCR in CP collected from an independent group of animals. This analysis confirmed the relative expression of these genes (data not shown), and indicated that genes with an expression level of 6.1, the lowest level in the array, are detectable by RT-qPCR. Altogether, it was inferred from the analysis that the array data represented genes expressed under basal physiological conditions.

**Figure 2:**
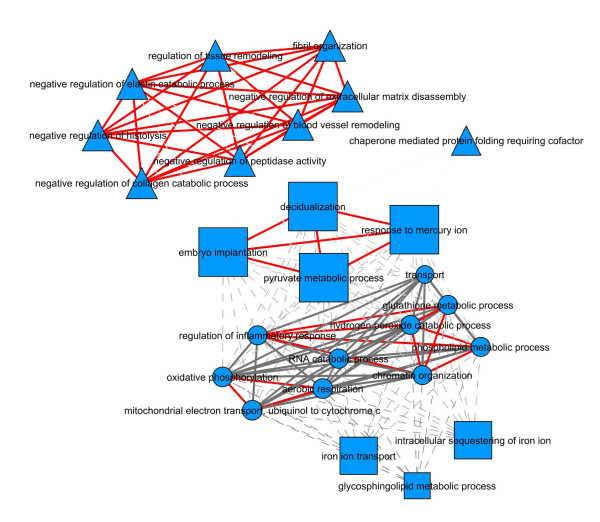
**Functional and network analysis of the most highly-expressed genes**. Analysis of the genes displaying expression levels above 13 clustered in particular functional networks. Different shapes represent different modules of biological functions. Dashed lines are relationships between nodes in different modules and solid lines are relationships between nodes in the same module. Dashed lines depict medium strong interactions (i.e. in between the median of the distribution and its upper quartile) and solid lines indicate the strongest interactions (i.e. superior to the upper quartile of their distribution). The interactional centrality of GO themes is illustrated by the size of the corresponding nodes. A red line denotes that the same gene is linked between all the biological process and gray lines denote different genes in each node.

Table 1 lists the fifty-nine most highly expressed genes (threshold 13.0 in the array). Analysis of the genes displaying expression levels above 13 were clustered in particular functional networks (Figure 2). Analysis of gene function networks identified 3 modules of interaction for the 59 mostly expressed genes. One included genes participating in pathways such as regulation of the tissue remodelling, fibril organization, negative regulation of processes such as extracellular matrix disassembly and blood vessel remodelling. Another, included genes of iron homeostasis, carbohydrate and lipid metabolism. A third module included genes involved in free radical homeostasis, regulation of the inflammatory response, mitochondrial metabolism and RNA processing. A table containing the various genes in each module is provided as Additional File 2 (Functional networks of the most highly-expressed genes in the choroid plexus).

**Table 1:** Most highly expressed genes found in the choroid plexus in normal physiological conditions.

Symbol	Definition	E.value
Ubb	ubiquitin B	15.0
Igf2	insulin-like growth factor 2	14.6
Rpl41	ribosomal protein L41	14.5
Cox4i1	cytochrome c oxidase subunit IV isoform 1	14.1
Clu	clusterin	14.1
Psap	prosaposin	14.0
Chchd10	coiled-coil-helix-coiled-coil-helix domain containing 10	14.0
Arl6ip1	ADP-ribosylation factor-like 6 interacting protein 1	13.9
Atp5b	ATP synthase, H+ transporting mitochondrial F1 complex. beta subunit	13.9
Cst3	cystatin C	13.9
Clic6	chloride intracellular channel 6	13.8
Ttr	transthyretin	13.8
1500015O10Rik	RIKEN cDNA 1500015O10 gene	13.7
Grim19	genes associated with retinoid-IFN-induced mortality 19	13.7
Cox8a	cytochrome c oxidase, subunit VIIIa	13.7
Gapd	glyceraldehyde-3-phosphate dehydrogenase	13.7
Atp5h	ATP synthase. H+ transporting, mitochondrial F0 complex. subunit d	13.7
Uba52	ubiquitin A-52 residue ribosomal protein fusion product 1	13.6
Uqcrh	ubiquinol-cytochrome c reductase hinge protein	13.6
Ckb	creatine kinase. brain	13.6
Cox6a1	cytochrome c oxidase. subunit VI a, polypeptide 1	13.6
Gpx4	glutathione peroxidase 4	13.6
Rps14	ribosomal protein S14	13.6
Ppia	peptidylprolyl isomerase A	13.6
Rplp1	ribosomal protein. large, P1	13.6
Aldh2	aldehyde dehydrogenase 2, mitochondrial	13.6
Rnaset2	ribonuclease T2	13.5
Rps27a	ribosomal protein S27a	13.5
Rps29	ribosomal protein S29	13.5
Dbi	diazepam binding inhibitor	13.5
Atp5j2	ATP synthase, H+ transporting. mitochondrial F0 complex, subunit f. isoform 2	13.5
Grina	glutamate receptor, ionotropic. N-methyl D-asparate-associated protein 1 (glutamate binding)	13.5
1110020P15Rik	RIKEN cDNA 1110020P15 gene	13.5
Atp1a1	ATPase, Na+/K+ transporting. alpha 1 polypeptide	13.5
Fth1	ferritin heavy chain 1	13.4
Ptgds	prostaglandin D2 synthase (brain)	13.4
Atp5g3	ATP synthase. H+ transporting, mitochondrial F0 complex. subunit c (subunit 9), isoform 3	13.4
Cd81	Cd81 antigen	13.4
Ndufa1	NADH dehydrogenase (ubiquinone) 1 alpha subcomplex. 1	13.3
Atp1b1	ATPase. Na+/K+ transporting, beta 1 polypeptide	13.2
Cd63	Cd63 antigen	13.2
Cox5b	cytochrome c oxidase. subunit Vb	13.2
Ubc	ubiquitin C	13.2
Sostdc1	sclerostin domain containing 1	13.2
Rps20	ribosomal protein S20	13.2
Ubl5	ubiquitin-like 5	13.1
Aplp2	amyloid beta (A4) precursor-like protein 2	13.1
Scd2	stearoyl-Coenzyme A desaturase 2	13.1
Rbp1	retinol binding protein 1. cellular	13.1
Ndufa4	NADH dehydrogenase (ubiquinone) 1 alpha subcomplex, 4	13.1
Rpl3	ribosomal protein L3	13.1
2010107E04Rik	RIKEN cDNA 2010107E04 gene	13.1
Cryab	crystallin, alpha B	13.0
Vdac1	voltage-dependent anion channel 1	13.0
Car12	carbonic anyhydrase 12	13.0
Rpl35	ribosomal protein L35	13.0
Cox7c	cytochrome c oxidase. subunit VIIc	13.0
Rps21	ribosomal protein S21	13.0
Ldh2	lactate dehydrogenase 2, B chain	13.0

**Figure 3:**
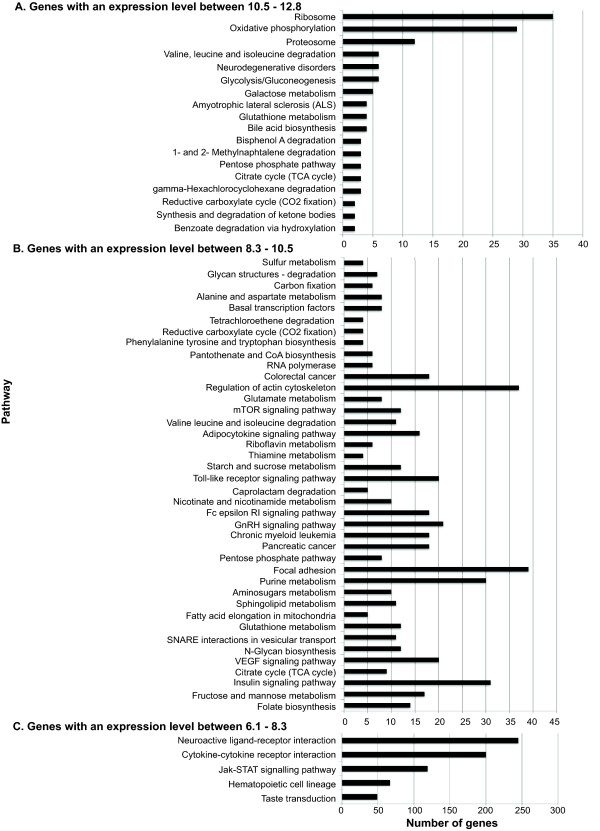
**Numbers of genes in different metabolic pathways expressed in the choroid plexus in normal physiological conditions**. Genes that have expression levels from 6.1-8.3 (A); from 8.3-10.5 (B) and from 10.5-12.8 (C).

Figure 3 identifies the molecular pathways encompassing genes with an expression level between 6.1-12.8. Of note, is the number of genes clustered in classes related to oxidative phosphorylation and ribosomal function, which is in agreement with the high secretory capacity of the CP. Interestingly, adhesion and regulation of actin cytoskeleton are also highly expressed, probably reflecting the barrier functions. In addition, it highlights genes belonging to various signalling pathways.

### Classes of genes expressed in the CP and their functions

**Figure 4:**
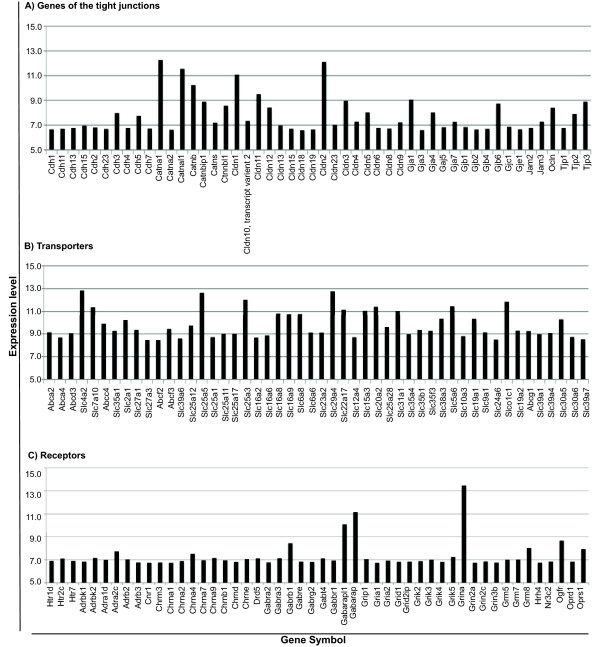
**Expression levels of choroid plexus genes that contribute to specific barrier properties**. Genes that encode for: tight junctions (A) transporters (B) and receptors (C).

The CP has traditionally been viewed as a tissue that provides the brain with secreted CSF, which through bulk flow, supplies the brain with some nutrients such as vitamin C, amino acids, nucleosides and transport proteins such as transthyretin. The CP is also known to facilitate the removal of products of brain metabolism such as free iodine, glutamate and amyloid beta peptide. On the other hand, the CP provides a barrier that restricts the passage of molecules and cells into and out of the brain. The present study provides solid evidence to support this view. In agreement we found that the CP transcriptome includes genes encoding for the tight junctions proteins (Figure 4A), for transporters namely for monocarboxylic acid (Slc16a9), glucose (Slc2a1), vitamin C (Slc23a2), thiamine (Slc19a2), zinc (such as Slc39a1) (Figure 4B) and for receptors of various classes of neurotransmitters such as dopamine, serotonin, opioids, cannabinoids, glutamate, histamine and GABA and also receptors for folate, benzodiazepine, estrogen and growth hormone (Figure 4C). Of notice, with respect to transporters, the CP highly expressed both the ABC (all 7 distinct sub-families: ABC1, MDR/TAP, MRP, ALD, OABP, GCN20, White) and the Slc families (Figure 4B). In addition to the MDR/TAP and MRP, the basal transcriptome also revealed the presence of the solute carrier organic anion families (OAT and OATP) of efflux transporters. Finally, the sodium-dependent system for the excitatory acidic amino acids, e.g., glutamate and aspartate, was also expressed in the CP and suggests a mechanism for net removal of potentially neurotoxic amino acids from the brain. The CP also expressed different genes that encode transporters for glucose, fatty acid, monocarboxylic acid, nucleoside, glycerol, cationic amino acid transporter y^+ ^system, iodide and neurotransmitter transporters (Figure 4B).

**Figure 5:**
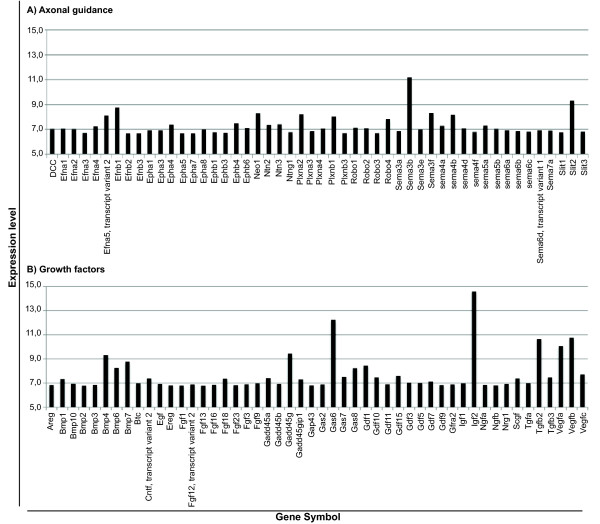
**Expression levels of relevant genes in the choroid plexus for brain development and for adult neurogenesis**. Genes belonging to axonal guidance molecules (A) and to the family of growth factors (B).

We chose to further highlight genes encoding for axonal guidance (Figure 5A), and for growth factors (Figure 5B) given their relevance for brain development and also for adult neurogenesis, as will be discussed later. These include insulin-like growth factor 2, transforming growth factor beta 2, vascular endothelial growth factor A and B, brain derived neurotrophic factor, ciliary neurotrophic factor transcript variant 2, fibroblast growth factors, betacellulin epidermal growth factor family member and its receptor ErbB-4, neuregulin 1, 3 and 4, amphiregulin, epiregulin and several members of the Wnt family (Additional File 1). With respect to axonal guidance, the CP basal transcriptome includes the four conserved families of guidance cues shown to have prominent developmental effects: netrins, semaphorins, SLIT and ephrins, as well as their respective receptors. Not surprisingly, among the most highly expressed genes are those encoding for major CSF proteins, such as transthyretin, prostaglandin D2 synthase, transferrin, insulin-like growth factor 2, clusterin (apolipoprotein J) and cystatin C (Table 1).

### CP transcriptome comparison with other mouse tissue transcriptomes

From the 62 tissues analysed, the CP transcriptome correlated with the transcriptome of the BBB endothelial cells more closely than with tissues from other brain regions or other peripheral tissues (data not shown).

We compared our CP array data with two others that are deposited in the GEO database, the GSE11443 and GSE3594. We observed that the correlation between our CP data was approximately 56% when compared to GSE11443 and 57% when compared to GSE3594. To test if the correlation we found between our study and the two others was acceptable, we also compared the correlation between the two other studies and found a correlation of 63%. Interestingly a similar correlation was found when the liver transcriptome from different studies was compared, which in this case had a correlation of 53%. Since the mostly expressed genes are those that best characterize a tissue, we also analysed if the fifty most highly-expressed genes in our study corresponded to the same fifty most highly-expressed genes in the two other CP studies. We observed that 34 and 20, from the GSE11443 and GSE3594 respectively, are common between the studies. Among the mostly expressed genes in these 3 studies are the genes encoding for *Arl6ip1, Ckb, Cox6a1, Cst3, Igf2, Ptgds, Rbp1, Rpl41, Slc4a2, Ttr *and *Ubc.*

## Discussion

Here we analysed and characterized the mouse CP transcriptome under normal basal physiological conditions. Unravelling the transcriptome signature of a tissue is a valuable tool for better understanding its functions in health and in disease. In this particular case, as part of the barriers that separate the brain from the periphery, it has become clear that the CP is more than an obstacle for molecule and cell trafficking into and out of the brain; it is an active participant in brain homeostasis.

Not surprisingly, we confirmed that among the genes most highly expressed were those encoding for proteins that are secreted into the CSF, such as transthyretin and transferrin. These are carriers for the ligands thyroxine and iron respectively, which are relevant for proper brain function during development and throughout adulthood. While CP transthyretin in itself does not seem essential for thyroid hormone to reach and distribute within the brain parenchyma [21], the available data does not exclude a role for the CP in thyroid hormone homeostasis. In fact, the thyroid gland is the sole site recognized for thyroid hormone synthesis. However, when the thyroid gland is ablated, it takes several months for the tissues to be depleted of thyroid hormones [22,23], which raises the possibility of extrathyroidal sites for thyroid hormone synthesis. The CP could be such a place, not only because it expresses the sodium-iodine symporter [24], which uptakes iodine against a concentration gradient, but also several peroxidases (glutathione peroxidase 4, 1, 3, 7, myeloperoxidase, thyroid peroxidase) as we now show in this study. Future investigation should address whether these peroxidases have the same ability as thyroid peroxidase in the thyroid to oxidise iodine and incorporate it into thyroglobulin (also present in the basal CP transcriptome) or other tyrosine containing protein.

Analysis of the basal transcriptome revealed genes involved in iron homeostasis such as transferrin; however, it is notable that participation in brain iron homeostasis seems to be particularly relevant in response to stimuli such as peripheral inflammation [11]. These observations highlight the ability of the CP to respond/adapt to physiological and disease states.

Several genes were shown to be expressed in the CP for the first time. These include those encoding for the opioid receptors, for which we found mRNA for all members of the family, the opioid receptor sigma 1 being the most highly expressed. Other studies, using autoradiographic techniques with selective ligands, failed to detect the mu and delta opioid receptors in rat CP brain [25,26], probably due to the lower sensitivity of the methodologies used. The present study also confirmed the presence of other neurotransmitter receptors previously described in the CP [27], such as the dopamine receptors. Again, this emphasizes an active role of the CP as a potential site for modulating response to neurotransmitters, which should be further investigated in the context of mood disorders.

We consider it of interest that the adult CP expresses genes encoding for several growth factors and axonal guidance molecules. While semaphorin 3f [2] and Slits 2 and 3 [28,29] were previously described in the developing CP, we described here for the first time the expression of various netrins and ephrins. The present study also adds to previously described growth and trophic factors [30,31], such as insulin-like growth factor II [32], glial cell line-derived neurotrophic factor [33], transforming growth factor alpha [34], fibroblast growth factor 2 [35] and vascular endothelial growth factor [36]. The expression of genes encoding for several members of the FGF family (those with higher expression value being FGF 18, 9 and 3), neuregulin, betacellulin and epiregulin should be highlighted. Betacellulin and its receptor ErbB-4 are highly expressed in neurons, implying important roles in neuronal cell functions; and inducing ErbB-4 activation promotes neurite outgrowth in PC12 cells [37]. Numerous genetic modelling studies in mice demonstrate that neuregulin 1 signalling is important in the development of normal neuronal connectivity. Of particular interest to this discussion are findings linking deficits in neuregulin 1-ErbB4 signalling to perturbations of synaptic transmission, myelination, and the survival of particular sets of neurons and glia [38]. It will be of interest to next investigate the participation of the CP in demyelinating diseases.

The CP expression of growth factors is also relevant in the context of neurogenesis. The subventricular zone is, together with the hippocampus, a site of adult neurogenesis. We showed here that the genes encoding for molecules that modulate adult neural stem cell proliferation and fate are transcribed in the CP. It is surprising that few studies so far have considered the CP as a relevant source of such mediators. The present study draws attention to the CP, as a modulator of the CSF composition, in the distribution of key molecules for neuronal differentiation.

Finally, the data we presented here confirms that the CP transcriptome displays a specific signature, different from all other tissues for which transcriptome data is available [39]. While one could anticipate resemblance with other secretory epithelia, such as the lung, the closest similarity is with that of the PECAM-1+ BBB microvascular endothelial cells [18]. This most likely reflects the fact that both have a barrier function, mediated by tight junctions; participate in the transport of molecules in and out of the brain, through several transporters and receptors; and in detoxifying processes, through several enzymes [31,40]. Despite these similarities, there are striking differences in gene expression between these two barriers, such as is the case for carbonic anhydrase 12, which is involved in CSF production (13.0 versus 4.4).

## Conclusions

In summary, the present work reveals that the CP is a site of active expression of several molecular pathways of relevance for brain homeostasis. Understanding its involvement in various physiological and disease states may provide novel clues to disease mechanisms, and also unravel novel targets for therapies against diseases of the central nervous system.

## List of abbreviations

BBB: blood-brain barrier; CP: choroid plexus; CSF: cerebrospinal fluid; CV: coefficient of variation; KEGG: Kyoto Encyclopedia of Genes and Genomes; MIAME: Minimum Information About a Microarray Experiment; ppMBMECs: PECAM-1+ blood-brain barrier microvascular endothelial cells; RT-qPCR: real time quantitative PCR.

## Competing interests

The authors declare that they have no competing interests.

## Authors' contributions

FM: conceived the study, participated in its design, preformed the experiments and collected, analysed and interpreted the data. JCS: conceived the study and participated in its design. GC: collected, analysed and interpreted the data. FG, RP, HB, and DG analysed and interpreted the data. NS: Conceived the study, participated in its design and discussed the findings. MCN: Conceived the study, participated in its design and discussed the findings. JAP: Conceived the study, participated in its design, discussed the findings and coordinated the study.

All authors have read and approved the final version of the manuscript.

## Supplementary Material

Additional file 1 **Expression levels of the genes transcribed in the basal choroid plexus**. The table lists the expression levels of all genes expressed in the basal CP transcriptome, after normalization as specified in the Methods section. Click here for file

Additional file 2 **Functional networks of the most highly-expressed genes in the choroid plexus**. The 59 most highly expressed genes were grouped in a 3-modular theme proximity network. The genes belonging to each module are specified. Click here for file
